# Insufficient labelling of allergen traces in foodstuffs – a survey for parents of children with food allergy

**DOI:** 10.1186/2045-7022-3-S3-P113

**Published:** 2013-07-25

**Authors:** B Kalb, V Trendelenburg, J Bellach, K Beyer

**Affiliations:** 1 Charité University Hospital, Berlin, Germany

## Background

Under applicable EU law, there´s an obligation for declaration of the 14 most common food allergens in packed foodstuffs, even if these allergens are contained in very low amounts. Concerning the declaration of allergen traces, however, there is no legally regulated obligation. Therefore it´s up to the food producers, if labelling of allergen traces takes place and how it is performed.

### Aim of the study

Main objective of our study is to evaluate, how common used food allergen labelling or the absence of these notes is interpreted by concerned parents in Germany.

## Methods

Based on a pilot study, using an open questionnaire, we developed a well standardised questionnaire, with which we interviewed 100 parents of children with food allergy (serologically diagnosed as well as according to a positive oral food challenge). The concerned parents were asked to evaluate different examples of lists of ingredients, with and without precautionary warnings, if these foodstuffs could be consumed by persons suffering from food allergies. The following warning phrases have been used: “Produced in a factory, where… is used“; “May contain traces of … “; “May contain “Schalenfrüchte” (german general term for certain nuts and seeds).“. … Contains….“

## Results

In general 60% of the respondent parents did not know, that allergen labelling of food traces is not legally mandatory regulated. If foodstuff is labelled with a warning note concerning allergen traces, 91% of the interviewed parents would not offer it to persons with food allergies. On the other hand, in absence of warning notes, 86% of the concerned parents considered that these “unlabelled” foodstuffs could be consumed by persons with food allergies. Differently worded precautionary warnings were associated with a differently high risk concerning cross contamination by 72% of the respondent parents.

## Conclusion

The risk of cross- contamination in foodstuffs has been differently interpreted, dependent on the chosen wording or the presence/absence of precautionary warning notes. Particularly serious is the fact, that in absence of warning notes concerning possible cross-contamination, the majority of the concerned parents considered these foodstuffs as harmless for persons with food allergies. Therefore our study shows the pressing need of a legally regulated, clear and consistent labelling of allergen traces in order to not compromise the health and safety of persons with food allergies.

## Disclosure of interest

None declared.

